# Activation-Induced Cell Death of Dendritic Cells Is Dependent on Sphingosine Kinase 1

**DOI:** 10.3389/fphar.2016.00094

**Published:** 2016-04-15

**Authors:** Anja Schwiebs, Olga Friesen, Elisabeth Katzy, Nerea Ferreirós, Josef M. Pfeilschifter, Heinfried H. Radeke

**Affiliations:** ^1^Department of General Pharmacology and Toxicology, Pharmazentrum Frankfurt/ZAFES, Clinic of the Goethe UniversityFrankfurt, Germany; ^2^Department of Clinical Pharmacology, Pharmazentrum Frankfurt, Clinic of the Goethe UniversityFrankfurt, Germany

**Keywords:** sphingosine-1-phosphate, S1P, activation-induced-cell-death, dendritic cells, sphingosine kinase 1, sphingosine kinase 2, FTY720P, Fingolimod

## Abstract

Sphingosine 1-phosphate (S1P) is an immune modulatory lipid mediator and has been implicated in numerous pathophysiological processes. S1P is produced by sphingosine kinase 1 (Sphk1) and Sphk2. Dendritic cells (DCs) are central for the direction of immune responses and crucially involved in autoimmunity and cancerogenesis. In this study we examined the function and survival of bone marrow-derived DCs under long-term inflammatory stimulation. We observed that differentiated cells undergo activation-induced cell death (AICD) upon LPS stimulation with an increased metabolic activity shortly after stimulation, followed by a rapid activation of caspase 3 and subsequent augmented apoptosis. Importantly, we highlight a profound role of Sphk1 in secretion of inflammatory cytokines and survival of dendritic cells that might be mediated by a change in sphingolipid levels as well as by a change in STAT3 expression. Cell growth during differentiation of Sphk1-deficient cells treated with the functional S1P receptor antagonist FTYP was reduced. Importantly, in dendritic cells we did not observe a compensatory regulation of Sphk2 mRNA in Sphk1-deficient cells. Instead, we discovered a massive increase in Sphk1 mRNA concentration upon long-term stimulation with LPS in wild type cells that might function as an attempt to rescue from inflammation-caused cell death. Taken together, in this investigation we describe details of a crucial involvement of sphingolipids and Sphk1 in AICD during long-term immunogenic activity of DCs that might play an important role in autoimmunity and might explain the differences in immune response observed in *in vivo* studies of Sphk1 modulation.

## Introduction

Sphingosine 1-phosphate (S1P) is a pleiotropic lipid mediator and has been implicated in numerous physiological processes and diseases that affect several organs in humans (Maceyka et al., 2011). S1P is generated from sphingosine by phosphorylation via sphingosine kinase 1 and 2 (Sphk1 and 2; Alemany et al., 2007; Spiegel and Milstien, 2007) and has a broad range of cellular functions which include cell growth, survival, and differentiation as well as lymphocyte trafficking and inflammation (Spiegel and Milstien, 2011; Arlt et al., 2014). S1P is also suspected to regulate cancerogenic conditions (Colie et al., 2009; Tabasinezhad et al., 2014; Zhang et al., 2016). Interestingly, functions of S1P are depending on its localization: Intracellular, it acts as a second messenger in a variety of cell signaling processes, while upon secretion it activates as an extracellular ligand five distinct G protein-coupled receptors (S1PR_1_–S1PR_5_) in a paracrine or autocrine manner (Van Brocklyn et al., 1998; Payne et al., 2002; Itagaki et al., 2007). Because of its diverse and potent bioactivity, the concentrations of S1P are tightly regulated by S1P-generating enzymes (Sphk1/2) and several S1P metabolizing enzymes, namely S1P phosphatases 1 and 2, lipid phosphate phosphatases 1–3 (Le Stunff et al., 2002) (Brindley and Pilquil, 2009) and S1P lyase which catalyzes an irreversible cleavage of S1P (Bandhuvula and Saba, 2007; Saba and de la Garza-Rodea, 2012).

S1P has been described in detail for its transactivating properties in cell proliferation induced by PDGF (Olivera and Spiegel, 1993), for the maintenance of islet viability (Lee et al., 2013), for its inhibition of histone deacetylases HDAC1/2 (Hait et al., 2009) and for its intracellular effects on the TRAF2/RIP1/NF-κB signaling pathway (Alvarez et al., 2010). We have shown that S1P regulates cytokine secretion of immune cells as it e.g., reduces IL-12p70 production shortly after lipopolysaccharide (LPS)—triggered toll-like-receptor (TLR)-4 stimulation of splenocytes and dendritic cells (Schroeder et al., 2011). Sphingosine 1-phosphate modulates antigen capture (Japtok et al., 2012) and several selective S1P1 receptor-modulating drugs are being investigated in clinical trials for the treatment of diverse autoimmune disorders (Zu Heringdorf et al., 2013). FTY720 (Fingolimod; Gilenya®) is an immune-modulatory prodrug which, after intracellular phosphorylation (FTYP) by sphingosine kinase 2 and subsequent export, mimics effects of the endogenous lipid mediator S1P via S1P1, S1P3-5 receptors. Thus, Fingolimod has been introduced to treat relapsing-remitting multiple sclerosis by inhibiting central memory T cell egress from secondary lymphoid organs (Brinkmann et al., 2010). However, Fingolimod has also direct influences on immune cell function (Ottenlinger et al., 2016) and is able to accumulate intracellularly (Schroder et al., 2015).

Dendritic cells (DCs) are key players of the immune system as they play a central role in lymphocyte activation and differentiation into T helper type 1 (Th1), Th2, Th17 as well as cytotoxic T effector cells by the expression of co-stimulatory molecules, chemokines, cytokines, and the presentation of MHC molecules on their surface (Iwasaki and Medzhitov, 2004; Siegemund et al., 2009; Arlt et al., 2014). With subtle differences between conventional and plasmacytoid DCs, in general these cells express all types of TLRs. TLR4 can be found on both, the plasma membrane of the cell and at internal, endosomal membranes (Gangloff, 2012). DCs are highly specialized for antigen (cross-) presentation endowed with limited acidification of their phagolysosomes to prevent complete proteolysis of antigens. While being essential for immune defense, in the presence of danger signals, such as an excess of apoptotic cells, the host-derived nucleic acids may activate these TLRs and propagate autoimmunity.

In order to understand the role of S1P metabolism for dendritic cell activity and survival, in this study we investigated S1P enzyme expression and sphingolipid metabolism during TLR4-dependent activation of DCs. Moreover, we analyzed cytokine production and dendritic cell viability over time and unraveled a connection between S1P metabolites, enzymes, and dendritic cell apoptosis that might play a role in inflammatory responses and autoimmunity.

## Materials and methods

### Isolation, differentiation, and stimulation of bone marrow cells

For isolation of bone marrow cells female WT C57BL/6 mice (Janvier, Saint Berthevin Cedex, France) and female Sphk1-deficient (Sphk1^−∕−^) mice [with C57BL/6 background, generated by Genoway, Lyon, France as described by Pushparaj et al. (2009)] bred at the local animal facility under specific pathogen-free conditions were used. All animal experiments were performed in accordance with the German animal welfare law and had been declared to the Animal Welfare Officer as the chairperson of the ethical oversight committee of the Goethe University Frankfurt/Main. The animal housing facility was licensed by the local authorities of the Regierungspraesidium Darmstadt (Az: 32.62.1). The methods used to euthanize the animals humanely were consistent with the recommendations of the AVMA Guidelines for the Euthanasia of Animals. After euthanasia of the mice, tibia, and femur were dissected, washed in ethanol and the ends of the bones were sliced. The bone marrow was rinsed with PBS (Thermo Fisher Scientific, Darmstadt, Germany) and erythrocytes were lysed by ACK buffer. Prior to cultivation the bone marrow cells were washed with RPMI 1640 GlutaMax medium (Thermo Fisher Scientific) supplemented with 10% FCS, 100 IU/ml penicillin, 100 μg/ml streptomycin, 10 mM HEPES (Sigma–Aldrich, Steinheim, Germany), 1 mM sodium pyruvate, and 50 μM 2-β-ME (Thermo Fisher Scientific). The cells were seeded at a density of 0.5 × 10^6^ cells/ml in culture medium supplemented with 40 ng/ml GM-CSF and cultured in a humidified incubator (5% CO_2_ and 37°C) for 7 days including one medium substitution. After this expansion phase differentiated immature bone marrow derived DCs (BM-DCs) were scraped and seeded at 1 × 10^6^ cells/ml in 12- or 6-well plates with 1 or 2 ml of culture medium without FCS and GM-CSF. After 1 h of incubation at 5% CO_2_ and 37°C the cells were stimulated with 1 μg/ml LPS from *Escherichia coli* O127:B8 (Sigma-Aldrich) and/or 1 μg/ml FTYP (kindly provided by V. Brinkmann, Novartis, Basel, Switzerland), 10 μM staurosporine (LC Laboratories, Woburn, Massachusetts), or left un-treated for the indicated time points. In cell differentiation studies, FTYP was applied daily to the cells within the expansion phase. After stimulation, a part of the harvested cell suspension was used for trypan blue (Sigma-Aldrich) staining. Upon centrifugation the cell pellet was used either for RNA isolation, protein extraction or lipid extraction. The supernatants were analyzed by mouse-specific ELISAs for IL-23, IL-10, and IL-6 (R&D Systems, Wiesbaden, Germany) according to the manufacturer's manual.

### Flow cytometry

To identify apoptotic cells, BM-DCs were resuspended in Annexin V binding buffer with Annexin V-FITC (ImmunoTools, Friesoythe; Germany) for 15 min in the dark. Hereafter, 7-AAD-PercP (eBioscience, Frankfurt am Main, Germany) was given to the cells (5 min), which were finally suspended in 400 μl Annexin V binding buffer. Data were acquired with a FACSCantoII flow cytometer (BD Biosciences, Heidelberg, Germany) and analyzed using the software FlowJo (TreeStar, Ashland, OR). Staurosporine treatment was used as a positive control.

### Metabolic activity

The XTT Cell Viability Assay (Thermo Fischer Scientific) was used according to the manufacturer's manual. In brief, the final XTT solution was added to cell culture wells with cells or medium only as a control. Upon 45 min incubation time at 5% CO_2_ and 37°C aliquots of the cells were analyzed in flat 96-well plates (Greiner, Frickenhausen, Germany) at 460 and normalized to 650 nm.

### Western blotting

For Western blot analysis 5 × 10^6^ BM-DCs were stimulated with 1 μg/ml LPS for the described time points. The pelleted cells were lysed in a buffer containing 10 mM HEPES-KOH, 10 mM KCl, 0.1 mM EDTA, 0.1 mM EGTA, 0.5 mM NaF, 1 mM Na3VO4, and 1 × complete™ protease inhibitor cocktail (Roche Diagnostics, Mannheim, Germany) by sonification on ice for 10 s. Total protein concentration was determined by BCA (Thermo Fisher Scientific), according to manufacturer's instructions. Whole cell extracts were used for detection of Sphk1 (Abnova, Heidelberg, Germany) and β-actin (Sigma-Aldrich). The cytosolic fraction was used after pelleting the nuclear fraction by centrifugation at 13,000 × g for 10 min at 4°C to detect caspase 3 (Cell Signaling Technology, Danvers, MA) or β-actin (Sigma Aldrich). According to the first antibodies, the second antibody anti-rabbit IgG (GE Healthcare, Little Chalfont, UK) has been used. The protein bands were detected by ECL (Thermo Fisher Scientific) following the manufacturer's protocol. Quantitative evaluation was performed by densitometry using Quantity one (Bio-Rad, Hercules, CA).

### Lipid extraction and sphingolipid analysis by LC-MS/MS

For the quantification of sphingolipids, cell pellets in methanol were spiked with an internal standard solution (500 ng/ml C17-Cer, 500 ng/ml Sph-d7, 500 ng/ml S1P-d7, and 500 ng/ml Saph-d7; Avanti Polar Lipids, Alabaster, USA). Afterwards, lipid extraction was performed twice using 35 μl of 1 M HCl, 480 μl of a salt-solution (0.74% KCl, 0.04% CaCl_2_, 0.034% MgCl_2_) and 600 μl of chloroform. Thereafter, the aqueous layer was collected, evaporated under a nitrogen stream and reconstituted in 100 μl methanol for the injection into the LC-MS/MS system.

For the chromatographic separation a Luna C18 column (Phenomenex, Aschaffenburg, Germany) was used. The two mobile phases (A) water:formic acid (100:0.1, v/v) and (B) acetonitrile: tetrahydrofuran:formic acid (50:50:0.1, v/v/v) were used with a flow rate of 0.3 ml/min. Ten microliter of each sample was injected into the system and the overall runtime was 16 min using the following gradient: 60% of (A)/40% of (B) for 0.6 min following a linear change within 3.9 min to 0% (A)/100% (B) and held for 6.5 min. A second linear gradient was applied within 0.5 min to 60% (A)/40% (B) and held for 4.5 min. MS/MS analyses were performed on an API4000 triple quadrupole system equipped with a TurboIonspray source (Electrospray Ionization; Applied Biosystems, Darmstadt, Germany) operated in positive mode. Two *m/z* transitions with a dwell time of 50 ms were recorded: for quantification (380.2 → 264.2) and for qualification (380.2 → 82.1), to exclude false positive results. For analysis the Analyst Software 1.5 (Applied Biosystems, Darmstadt, Germany) was used. Linearity of the calibration curve was proven from 0.05 to 250 ng/ml. The coefficient of correlation was at least 0.99. Variations in preciseness were less than 15% over the range of calibration.

### Quantitative real-time PCR

Total RNA of pelleted cells was extracted using the peqGOLD Total RNA Kit (peqlab, Erlangen, Germany) as recommended by the manufacturer. RNA concentration was measured using the Nano-Drop 1000 (Thermo Scientific) analyzer and was adjusted to 1 μg/μl for first-strand cDNA synthesis using the high-capacity cDNA reverse transcription kit (Life Technologies, Carlsbad, CA). TaqMan® gene expression assays (Life Technologies) were applied for Sphk1/2 and for the housekeeping genes CsnK2a2 and Fbxo38, which were purchased from Primer design (Southampton, United Kingdom). The Precision FAST Mastermix (Primer Design) was used and quantitative real-time PCR was run at 95°C for 2 min and 40 times at 95°C for 5 s, 60°C for 20 s (7500 Fast Real-Time PCR System, Applied Biosystems). Data was evaluated using the mean of the two housekeeping genes as a reference.

### Statistics

The software GraphPad Prism 5.0 (La Jolla, CA) was used to enter data, display graphs, and perform statistics by 1 way or 2 way ANOVA with Bonferroni posttest or paired Student's *t*-test. Data are represented as means ± SD and significant values are symbolized as asterisks (^*^/^**^/^***^) and hash keys (#, ##, ###), which represent *P*-values of ≤0.05/≤0.01/≤ 0.001.

## Results

### Activation-induced cell death upon LPS stimulation

**Figure 1:**
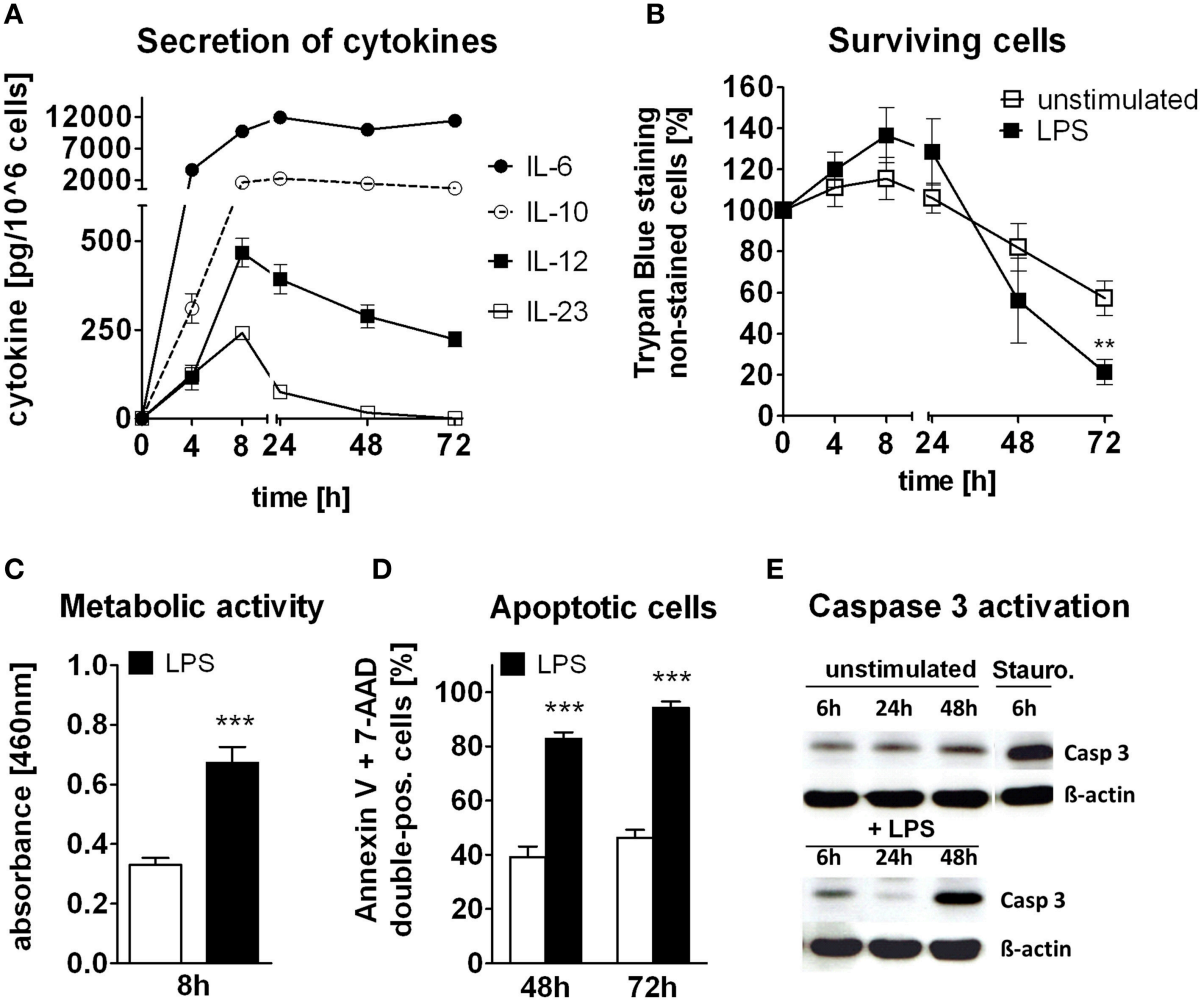
**Function and survival of TLR4 stimulated DCs**. GM-CSF differentiated cells have been stimulated with LPS for 0–72 h, **(A)** Quantification of cytokines in the supernatant of stimulated cells over time, **(B)** Trypan Blue staining of unstimulated and LPS-stimulated cells over time, **(C)** XTT-test for metabolic activity in unstimulated and 8 h LPS-stimulated cells, **(D)** FACS Annexin V and 7-AAD staining for apoptotic cells after 48 and 72 h of LPS-stimulation and in unstimulated cells, **(E)** Western Blot analysis for ß-actin (38 kDa) and cleaved caspase 3 (17 kDa) after 6, 24, and 48 h of LPS-stimulation as well as in unstimulated cells and staurosporin-treated cells as control. Time points of one group have been assembled on the same blot. Data represent mean ± SD, *n* ≥ 4 for each group, measured in duplicates for **(A–C)**; ^*^p ≤ 0.05, ^**^p ≤ 0.01, ^***^p ≤ 0.001.

Bone marrow-derived DCs were stimulated with LPS over 72 h and secretion of a variety of cytokines was determined (Figure 1A). High amounts of IL-6 and IL-10 were continuously secreted into the medium, whereas IL-12 and IL-23 reached a peak secretion 8 h after stimulation. Cell staining revealed that cells undergo proliferation within the first 24 h upon inflammatory stimulation (Figure 1B) Moreover, in this early activation period cells exert a higher metabolic activity (Figure 1C). After 24 h of stimulation, cell count declined. However, strikingly, stimulated cells die faster than normal cells. Annexin V/7-AAD staining revealed a higher frequency of apoptotic cells after LPS stimulation at late time points compared to control cells (Figure 1D). Whereas, half of the resting cells were still alive, 90% of activated DCs underwent apoptosis at the end of the cultivation. Indicating the apoptotic pathway, caspase 3 was highly activated 48 h after LPS stimulation in comparison to unstimulated cells (Figure 1E). Notably, in TLR4-stimulated cells, activation of caspase 3 was down regulated during the initial 24 h of the hyper-activation phase compared to baseline levels, whereas protein levels did not change in untreated cells.

### The Sphk1-S1P axis controls activation induced cell death (AICD)

**Figure 2:**
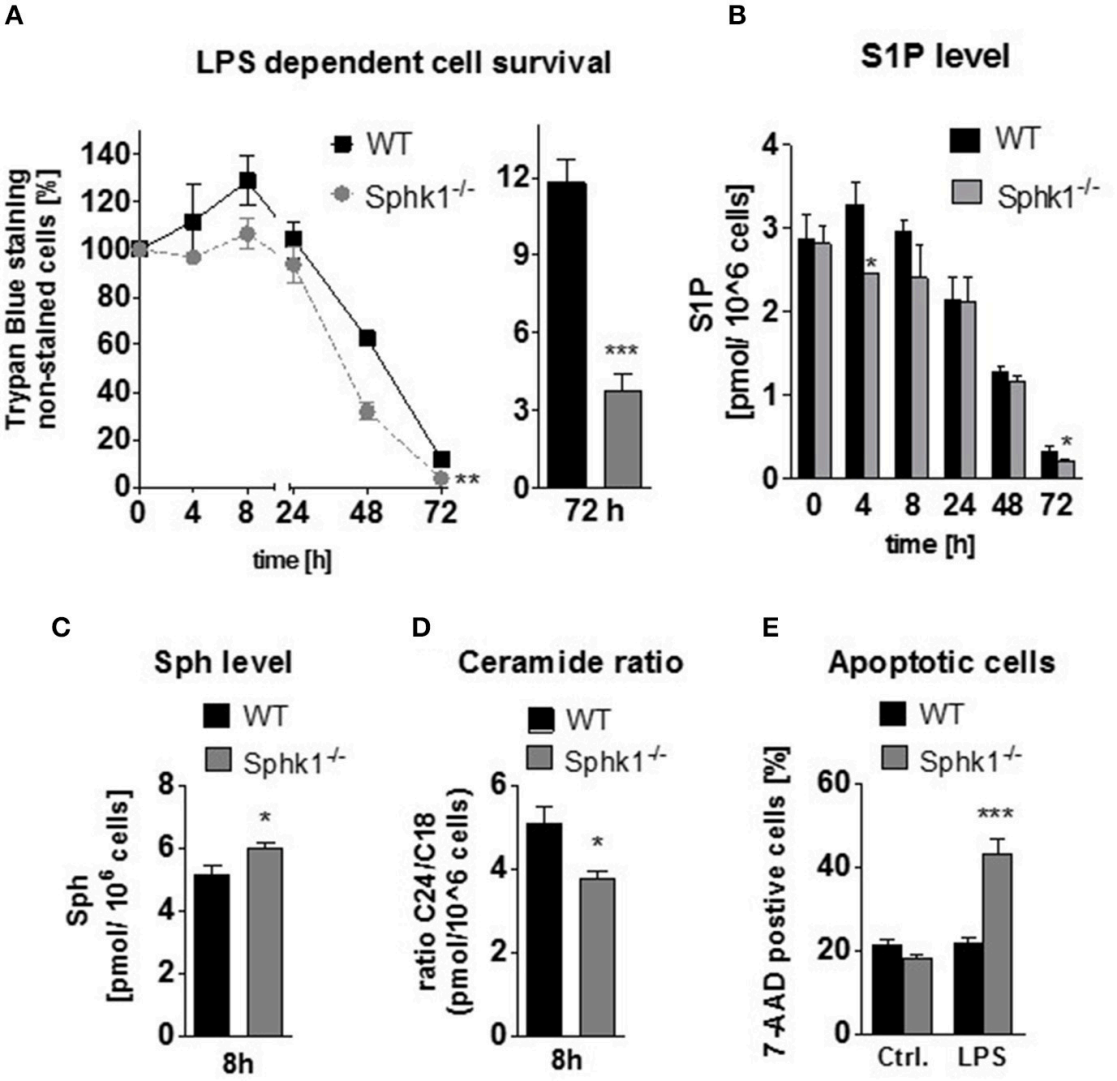
**Survival of sphingosine kinase 1 knockout BM-derived DCs**. GM-CSF-differentiated wild type and Sphk1-deficient cells have been stimulated with LPS for 0–72 h **(A)** Trypan Blue staining of LPS-stimulated cells over time, **(B)** LCMS/MS based determination of absolute intracellular sphingosine 1 phosphate level in wild type and Sphk1 deficient cells stimulated with LPS over time, **(C,D)** LC-MS/MS based determination of intracellular sphingosine, ceramide 24, and ceramide 18 levels in wild type and SphK1-deficient cells following 8 h of LPS stimulation, **(E)** FACS analysis of 7-AAD stained wild type and Sphk1-deficient cells upon 20 h of LPS stimulation and in unstimulated control cells. Data represent mean ± SD, *n* ≥ 4 for each group; ^*^p ≤ 0.05, ^**^p ≤ 0.01, ^***^p ≤ 0.001.

Since it is well-accepted, that S1P is an anti-apoptotic factor, we examined dendritic cell survival and function in the absence of the S1P-generating enzyme Sphk1. Interestingly, upon LPS stimulation, Sphk1-deficient BM-DCs do not undergo hyper-activation in the first 24 h and die faster, leaving alive only one third of the cells after 72 h (Figure 2A). Basal S1P levels were not significantly different between non-stimulated wild type and Sphk1-knockout cells. However, early after stimulation, S1P levels were significantly reduced in knockout cells vs. wild type cells (Figure 2B). Indeed, S1P levels tend to increase in wild type cells during the hyper-activation period whereas they rather decrease in Sphk1-deficient cells. From 2 h after stimulation, S1P level start to drop massively in both cell types. In accordance with this finding, sphingosine levels were increased early after stimulation in Sphk1-deficient cells (Figure 2C). Further analysis of downstream lipids revealed a decrease of the ceramide 24/ceramide 18 (Cer24/Cer18) quotient indicating a higher proliferative activity in wild type cells than in knockout cells (Figure 2D). Furthermore, more apoptotic cells were determined in Sphk1-deficient cells compared to wild type cells after TLR4 stimulation (Figure 2E).

**Figure 3:**
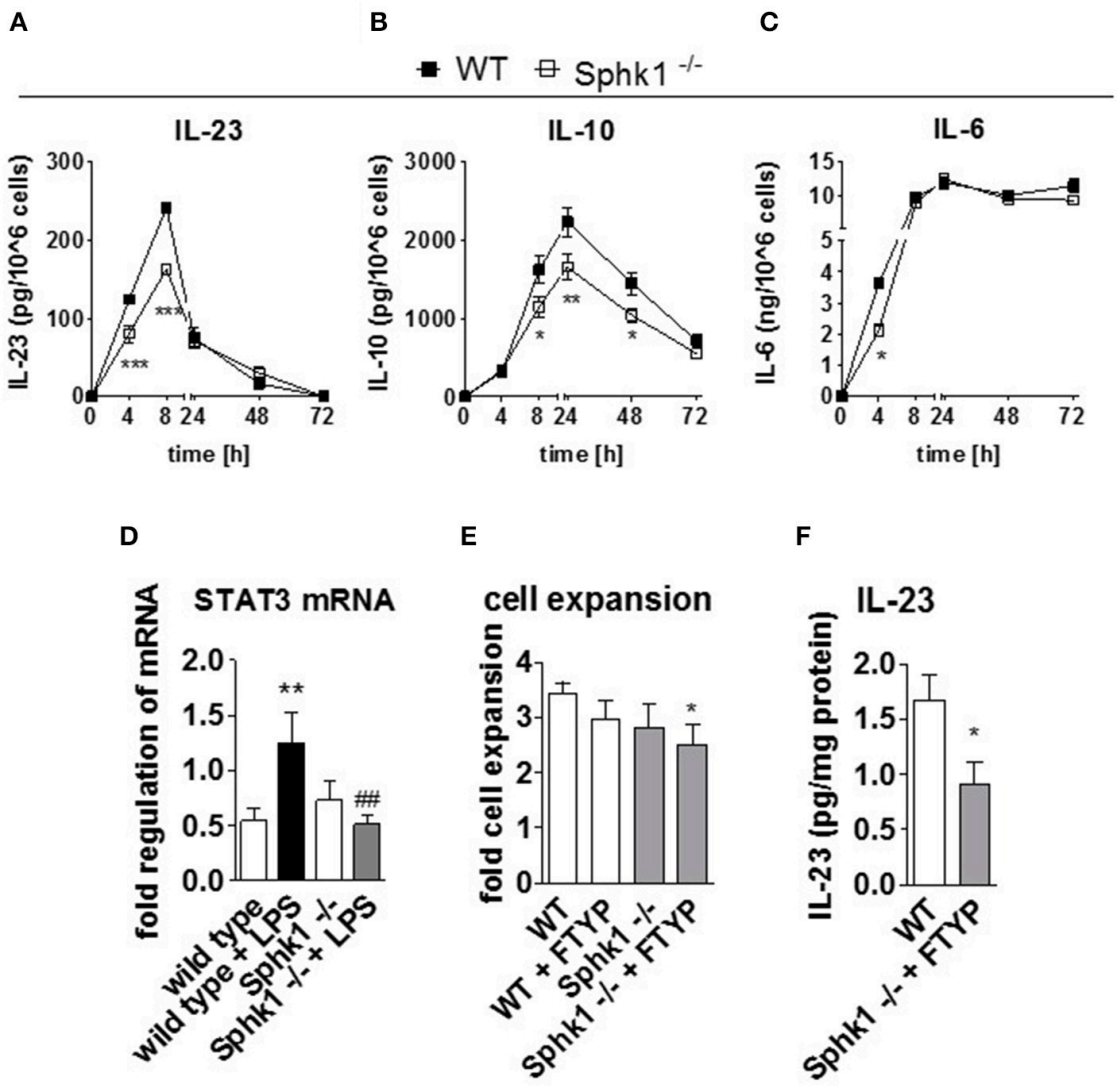
**Role of the Sphk1-S1P receptor module for function and expansion of DCs. (A–D)** GM-CSF-differentiated wild type and Sphk1-deficient cells have been stimulated with LPS for 0–72 h. **(A–C)** Quantification of cytokines in supernatants of stimulated wild type and Sphk1-deficient cells over time, **(D)** Determination of STAT3 mRNA level by quantitative real time PCR in wild type and Sphk1-deficient cells after 8 h of LPS stimulation and in unstimulated cells, **(E)** bone marrow cells from wild type and Sphk1-deficient mice have been differentiated with GM-CSF in the absence or presence of FTYP for 7 days and cell expansion has been monitored by cell count. **(F)** IL-23 secretion upon 20 h of LPS stimulation in GM-CSF differentiated wild type cells and GM-CSF + FTYP differentiated Sphk1-deficient cells. Data represent mean ± SD, *n* ≥ 4 for each group, measured in duplicates; ^*^p ≤ 0.05, ^**^p ≤ 0.01, ^***^p ≤ 0.001, ^*##*^*p* ≤ 0.01.

Analysis of interleukins revealed a decrease of IL-23, IL-10, and IL-6 level in an early phase after stimulation in Sphk1-deficient cells (Figures 3A–C). In line with a reduced IL-23 and IL-6 secretion, also early expression of STAT3 was low in cells lacking Sphk1, as opposed to increased mRNA levels in LPS stimulated wild type DCs (Figure 3D). Interestingly, Sphk1-deletion also affected BM-DC expansion during the GM-CSF differentiation phase (Figure 3E). Treatment with FTYP reduced cell growth significantly compared to wild type cells. Also, in the latter cells LPS stimulation after the differentiation phase resulted in a reduction of IL-23 secretion by 50% (Figure 3F).

### Regulation of Sphk1 levels in activated DCs

**Figure 4:**
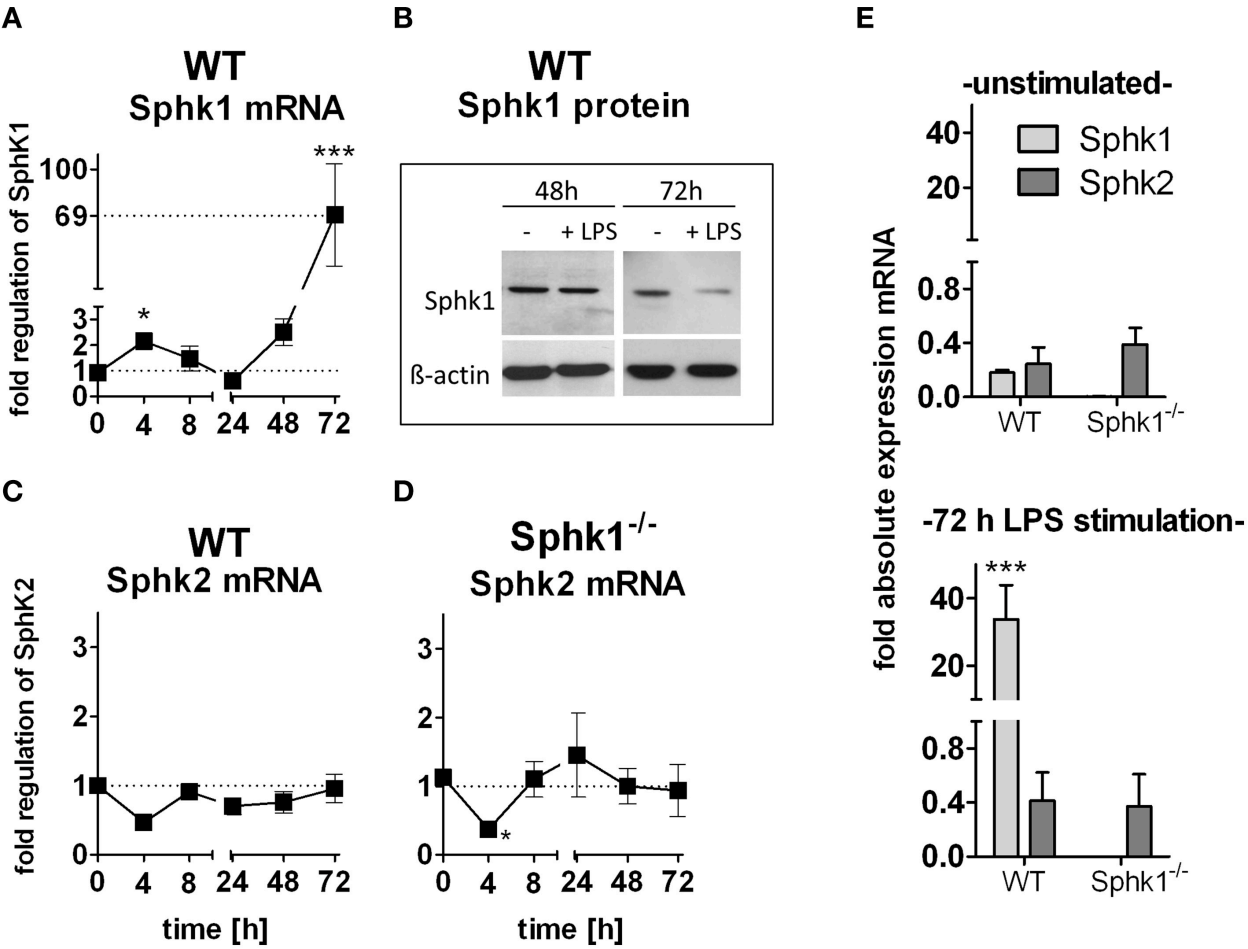
**Involvement of sphingosine kinases 1 and -2 in DC activation**. GM-CSF differentiated wild type and Sphk1-deficient cells have been stimulated with LPS for 0–72 h. **(A,C,D)** Determination of Sphk2 and Sphk1 mRNA level by quantitative real time PCR in wild type and Sphk1-deficient cells after LPS stimulation over time normalized to level of unstimulated cells which was set to 1 at every time point (dotted line). **(B)** Western Blot analysis of Sphk1 (51 kDa) and ß-actin (38 kDa) protein levels upon 48 and 72 h LPS stimulation and in unstimulated wild type cells. **(E)** Determination of absolute Sphk1 and Sphk2 mRNA level normalized to housekeeping genes (set to 1) by quantitative real time PCR in wild type and Sphk1 deficient cells upon 72 h of LPS stimulation. Data represent mean ± SD, *n* ≥ 5 for each group, measured in duplicates for **(A–E)**; ^*^p ≤ 0.05, ^***^p ≤ 0.001.

Expression levels of Sphk1 slightly increased during early stimulation in immune cells, however, levels rose massively by about 70-fold in late DCs (Figure 4A). Notably, this massive switch on of expression was not translated into protein levels, which were rather decreased after 72 h compared to those after 48 h of LPS-stimulation (Figure 4B).

Since a compensatory regulation of Sphk1 and Sphk2 was proposed, expression of both kinases was assessed on mRNA level. During the early peak of Sphk1 expression at 4 h, the Sphk2 level tended to drop at the same time (Figure 4C), however no significant downregulation of Sphk2 mRNA was found during the massive upregulation of Sphk1 mRNA in late DCs. Interestingly, in Sphk1-deficient cells the same early drop in Sphk2 mRNA concentration was found (Figure 4D) although no Sphk1 is expressed in those cells (data not shown). Absolute expression levels of both enzymes where not different under basal condition in wild type cells (Figure 4E). While Sphk1 was massively upregulated under stimulation, Sphk2 showed no change in expression levels.

## Discussion

Professional antigen presenting cells like DCs are pivotal in the recognition of foreign bacterial and viral components and the subsequent initiation of innate as well as of adaptive immune responses providing differentiation and activation of T cell subsets. Changes in the function and developmental status of DCs are induced by inflammatory stimuli like cytokines and bacterial products or by the cross-linking of surface molecules (Winzler et al., 1997). In this study we showed that after reception of such a signal, namely LPS, DCs accomplish maturation by undergoing hyper-activation and proliferation within the first 24 h. Notably, compared to immature DCs, the caspase 3 associated apoptotic pathway was diminished within this phase allowing DCs to proliferate.

DCs have relatively short half-lives, nevertheless, during this time, they are able to achieve successful antigen presentation to T cells (Nopora and Brocker, 2002). Immature DCs of the spleen are replaced within 3–4 days (Kamath et al., 2000) suggesting a short DC lifespan regulated by programmed apoptotic cell death. Granucci et al. described a regulation of modulators of life cycle and apoptosis accompanying DC activation (Granucci et al., 2001a,b). In our study, shortly after hyper-activation phase, DCs underwent a rapid caspase 3 mediated apoptosis pathway in which the majority of the cells were dead after 3 days while more than half of the immature cells were still alive under the same conditions. Hence, LPS-stimulated DCs undergo activation-induced cell death (AICD). The death of activated DCs may be an important contributor to the resolution of immune responses and to prevent the development of autoimmunity (Chen and Wang, 2010). Autoimmunity induced by DCs might be caused by increased DC activity, thus, apoptosis of DCs, upon completion of their task of antigen presentation, appears to serve as a negative immunoregulatory mechanism that may be crucial in controlling the magnitude of immune reactions against a given antigen (Hildeman et al., 2007; Stranges et al., 2007). Supporting this hypothesis, in our study LPS-activated DCs underwent faster apoptosis compared to their non-stimulated mates. In parallel to AICD of DCs we found S1P levels are tightly regulated indicating an association between sphingolipid homeostasis and cell survival.

Dysregulation of S1P concentrations are characteristic for an increasing number of diseases with disturbed immune modulation, especially autoimmune diseases (Bandhuvula and Saba, 2007; Brinkmann et al., 2010; Lai et al., 2011). Hence, it is of great importance to study the influence of S1P metabolism in association with survival and function of DCs.

As soon as S1P levels started to rise, DCs became active. The cell proliferation and survival seemed to follow intracellular S1P balances, since shortly after S1P concentrations were dropping, cells underwent apoptosis and survival started to decline. Strengthening this hypothesis, we found that Sphk1-deficient cells did not undergo hyper-activation in congruence with their S1P levels that did rather decrease than increase right after stimulation. As stated by Hartmann et al., also ceramides and especially ceramide chain length is decisive for cell fate (Hartmann et al., 2013). While long chain ceramides are proliferative, shorter ceramides are pro-apoptotic. The C24/C18 ratio describes the proliferative activity of the cells and was clearly reduced in Sphk1-deficient DCs. Accordingly, more apoptotic cells were found in Sphk1-deficient cells indicating that lower S1P levels decrease the activation responsiveness of DCs. Schwalm et al. and others showed that upon induction of Sphk2-deficiency, Sphk1 mRNA levels are upregulated in a compensatory manner and thus proliferative activity of e.g., renal mesangial cells or cancer cells is increasing (Liang et al., 2013; Schwalm et al., 2015). Vice versa however, in our bone marrow derived DCs, no significant compensatory regulation of Sphk2 mRNA was observed neither in Sphk1-deficient cells nor in the late apoptotic wild type cells where Sphk1 mRNA was massively accumulating. This might suggest that Sphk1-mediated S1P production is the key player in dendritic cell fate. Liang et al. and Lee et al. linked the proliferative activity of Sphk2-deficiency and S1PR_1_ overexpression to persistent STAT3 activation in tumor cells (Lee et al., 2011; Liang et al., 2013). Interestingly, in Sphk1-deficient DCs, activation of STAT3 expression was missing upon LPS-stimulation while in wild type cells levels started to rise very early after DC activation. Also, the secretion of the STAT3-targeting IL-23 was reduced in Sphk1-deficient cells especially during the missing early phase of DC hyper-activation. This reduction of IL-23 secretion of Sphk1 knockout cells suggests moreover, that the ability to induce a Th17 response could be suppressed. Indeed it was shown that Sphk1 knockout mice have fewer Th17 cells reducing TNFα induced inflammation (Baker et al., 2010). While, of the two additional cytokines known to be induced and at least partially signaling through STAT3, the more systemically functioning IL-6 was initially reduced but remained nearly unaltered in summary, IL-10 was significantly dampened, which may have an influence of subsequent Th1 response. Indeed, it was found that INFγ production by T lymphocytes was reduced upon Sphk1 inhibition indicating a suppression of the Th1 polarization (Jung et al., 2007).

Confirming our *in vitro* results showing a reduced DC activation, survival, and a possible reduced T-cell response provoked by a decrease in cytokine secretion in Sphk1-deficient cells, a reduced inflammatory response was shown in several recent *in vivo* studies using Sphk1 mice. In a mouse model of bacterial pathogen infection a reduced immune cell infiltration was found in Sphk1 deficient mice due to reduced S1P levels (Yu et al., 2016). Similarly, a reduced host defense against fungal pathogens was elucidated in Sphk1 knockout mice (Farnoud et al., 2015). In a model of allergic asthma it was shown that Sphk1 inhibition with a specific inhibitor decreased pulmonary inflammation (Price et al., 2013). Kawamori et al. showed that Sphk1 knockout mice were less prone to DSS/AOM induced colitis and associated colon cancerogenesis (Kawamori et al., 2009), whereas clinical data demonstrated that Sphk1 is overexpressed in colorectal tumors (Liang et al., 2013). Beach and colleagues showed a significant reduction of tumor growth and metastasis in an ovarian cancer model in Sphk1 knockout mice (Beach et al., 2015). Thus, our *in vitro* data in DCs may provide a molecular explanation of the prominent role of Sphk1 in immune response and even cancerogenesis.

Reduced Sphk1-S1P signaling also decreased dendritic cell growth during expansion phase as administration of FTYP to Sphk1-deficient cells diminished cell growth and IL-23 secretion compared to wild type cells. Hence, the Sphk1-S1P axis seems to be decisive for the distinct processes during the life circle of DCs, i.e., expansion, activation, and apoptosis. At the same time Sphk1 expression is tightly regulated in activated DCs. It was shown before, that LPS-stimulation of splenocytes increases the expression of Sphk1 by about tenfold within the first 2 h with a sharp decline thereafter (Schroeder et al., 2011). In early bone marrow-derived DCs a slight peak of Sphk1 mRNA was detected as well, but more interestingly, we determined a massive rise in Sphk1 mRNA in late apoptotic DCs. This upregulation might be a compensatory mechanism resulting from the loss of intracellular S1P levels, thus it seems to represent a rescue mechanism to avoid the apoptotic machinery. It might also be a reaction to the actual loss of Sphk1 protein in late DCs. In contrast, Sphk2 mRNA regulation over time appeared to be uncoupled from cell survival regulation by Sphk1 and S1P. As a consequence of our new observations it is of great interest to resolve the question how intracellular S1P levels are reduced over time. Possible mechanisms include the dephosphorylation to sphingosine, irreversible degradation e.g., by S1P lyase or export out of the cell. S1P transport has been ascribed to different ABC-transporters and spinster (SPNS2) in several cell types (Kobayashi et al., 2009; Nieuwenhuis et al., 2009; Hisano et al., 2012; Liu et al., 2012). However, it is still incompletely understood which transporters are expressed in bone marrow derived DCs and how much these plasma membrane transporters contribute to intracellular amphiphilic S1P regulation in deep, double lipid layer-separated compartments.

The key finding of this investigation is the closely intertwined regulatory relationship of basic cellular sphingolipid metabolism with highly specialized function of bone marrow-derived DCs. The most significant observation and conclusion that may be drawn is that potentially (i) pro-survival S1P supported initiation of an inflammatory reaction within the first 24 h whereas (ii) at later time points (48–72 h) a dangerous survival of continuously pro-inflammatory DCs was prevented by sphingolipid-related cell death. The latter finding of a connection between AICD and sphingolipid metabolism in DCs has not been shown before and might be relevant for a better understanding of the molecular mechanisms that regulate inflammatory responses, autoimmunity, and anti-cancer immunity.

## Author contributions

AS, OF, and EK performed the experiments. NF performed sphingolipid analytics. JP supplied basic lab equipment. AS designed the figures and performed statistics. AS and HR designed and wrote the manuscript. HR supervised all experiments.

## Funding

This work was supported by a grant of the Deutsche Forschungsgemeinschaft for the cooperative SFB1039 to project B03 of HHR, SFB1039 to project Z01 and moreover by DFG SPP1267.

### Conflict of interest statement

The authors declare that the research was conducted in the absence of any commercial or financial relationships that could be construed as a potential conflict of interest.

## References

[B1] Alemany R. van Koppen C. J. Danneberg K. Ter Braak M. Meyer Zu Heringdorf D. (2007). Regulation and functional roles of sphingosine kinases. Naunyn Schmiedebergs. Arch. Pharmacol. 374, 413–428. 10.1007/s00210-007-0132-3 17242884

[B2] Alvarez S. E. Harikumar K. B. Hait N. C. Allegood J. Strub G. M. Kim E. Y.. (2010). Sphingosine-1-phosphate is a missing cofactor for the E3 ubiquitin ligase TRAF2. Nature 465, 1084–1088. 10.1038/nature09128 20577214 PMC2946785

[B3] Arlt O. Schwiebs A. Japtok L. Rüger K. Katzy E. Kleuser B.. (2014). Sphingosine-1-phosphate modulates dendritic cell function: focus on non-migratory effects *in vitro* and *in vivo*. Cell. Physiol. Biochem. 34, 27–44. 10.1159/000362982 24977479

[B4] Baker D. A. Barth J. Chang R. Obeid L. M. Gilkeson G. S. (2010). Genetic sphingosine kinase 1 deficiency significantly decreases synovial inflammation and joint erosions in murine TNF-alpha-induced arthritis. J. Immunol. 185, 2570–2579. 10.4049/jimmunol.1000644 20644167 PMC2942019

[B5] Bandhuvula P. Saba J. D. (2007). Sphingosine-1-phosphate lyase in immunity and cancer: silencing the siren. Trends Mol. Med. 13, 210–217. 10.1016/j.molmed.2007.03.005 17416206

[B6] Beach J. A. Aspuria P. P. Cheon D. J. Lawrenson K. Agadjanian H. Walsh C. S.. (2015). Sphingosine kinase 1 is required for TGF-beta mediated fibroblastto-myofibroblast differentiation in ovarian cancer. Oncotarget 7, 4167–4182. 10.18632/oncotarget.6703 26716409 PMC4826197

[B7] Brindley D. N. Pilquil C. (2009). Lipid phosphate phosphatases and signaling. J. Lipid Res. 50 (Suppl.), S225–S230. 10.1194/jlr.R800055-JLR200 19066402 PMC2674702

[B8] Brinkmann V. Billich A. Baumruker T. Heining P. Schmouder R. Francis G.. (2010). Fingolimod (FTY720): discovery and development of an oral drug to treat multiple sclerosis. Nat. Rev. Drug Discov. 9, 883–897. 10.1038/nrd3248 21031003

[B9] Chen M. Wang J. (2010). Programmed cell death of dendritic cells in immune regulation. Immunol. Rev. 236, 11–27. 10.1111/j.1600-065X.2010.00916.x 20636805 PMC3282617

[B10] Colie S. Van Veldhoven P. P. Kedjouar B. Bedia C. Albinet V. Sorli S. C.. (2009). Disruption of sphingosine 1-phosphate lyase confers resistance to chemotherapy and promotes oncogenesis through Bcl-2/Bcl-xL upregulation. Cancer Res. 69, 9346–9353. 10.1158/0008-5472.CAN-09-2198 19934311

[B11] Farnoud A. M. Bryan A. M. Kechichian T. Luberto C. Del Poeta M. (2015). The granuloma response controlling cryptococcosis in mice depends on the sphingosine kinase 1-sphingosine 1-phosphate pathway. Infect. Immun. 83, 2705–2713. 10.1128/IAI.00056-15 25895971 PMC4468535

[B12] Gangloff M. (2012). Different dimerisation mode for TLR4 upon endosomal acidification? Trends Biochem. Sci. 37, 92–98. 10.1016/j.tibs.2011.11.003 22196451 PMC3323831

[B13] Granucci F. Vizzardelli C. Pavelka N. Feau S. Persico M. Virzi E.. (2001a). Inducible IL-2 production by dendritic cells revealed by global gene expression analysis. Nat. Immunol. 2, 882–888. 10.1038/ni0901-882 11526406

[B14] Granucci F. Vizzardelli C. Virzi E. Rescigno M. Ricciardi-Castagnoli P. (2001b). Transcriptional reprogramming of dendritic cells by differentiation stimuli. Eur. J. Immunol. 31, 2539–2546. 10.1002/1521-4141(200109)31:9&amp;#60;2539::AID-IMMU2539&amp;#62;3.0.CO;2-9 11536151

[B15] Hait N. C. Allegood J. Maceyka M. Strub G. M. Harikumar K. B. Singh S. K.. (2009). Regulation of histone acetylation in the nucleus by sphingosine-1-phosphate. Science 325, 1254–1257. 10.1126/science.1176709 19729656 PMC2850596

[B16] Hartmann D. Wegner M. S. Wanger R. A. Ferreiros N. Schreiber Y. Lucks J.. (2013). The equilibrium between long and very long chain ceramides is important for the fate of the cell and can be influenced by co-expression of CerS. Int. J. Biochem. Cell Biol. 45, 1195–1203. 10.1016/j.biocel.2013.03.012 23538298

[B17] Hildeman D. Jorgensen T. Kappler J. Marrack P. (2007). Apoptosis and the homeostatic control of immune responses. Curr. Opin. Immunol. 19, 516–521. 10.1016/j.coi.2007.05.005 17644328 PMC4127626

[B18] Hisano Y. Kobayashi N. Yamaguchi A. Nishi T. (2012). Mouse SPNS2 functions as a sphingosine-1-phosphate transporter in vascular endothelial cells. PLoS ONE 7:e38941. 10.1371/journal.pone.0038941 22723910 PMC3379171

[B19] Itagaki K. Yun J. K. Hengst J. A. Yatani A. Hauser C. J. Spolarics Z.. (2007). Sphingosine 1-phosphate has dual functions in the regulation of endothelial cell permeability and Ca2+ metabolism. J. Pharmacol. Exp. Ther. 323, 186–191. 10.1124/jpet.107.121210 17626797

[B20] Iwasaki A. Medzhitov R. (2004). Toll-like receptor control of the adaptive immune responses. Nat. Immunol. 5, 987–995. 10.1038/ni1112 15454922

[B21] Japtok L. Schaper K. Baumer W. Radeke H. H. Jeong S. K. Kleuser B. (2012). Sphingosine 1-phosphate modulates antigen capture by murine Langerhans cells via the S1P2 receptor subtype. PLoS ONE 7:e49427. 10.1371/journal.pone.0049427 23145172 PMC3493526

[B22] Jung I. D. Lee J. S. Kim Y. J. Jeong Y. I. Lee C. M. Baumruker T.. (2007). Sphingosine kinase inhibitor suppresses a Th1 polarization via the inhibition of immunostimulatory activity in murine bone marrow-derived dendritic cells. Int. Immunol. 19, 411–426. 10.1093/intimm/dxm006 17307797

[B23] Kamath A. T. Pooley J. O'Keeffe M. A. Vremec D. Zhan Y. Lew A. M.. (2000). The development, maturation, and turnover rate of mouse spleen dendritic cell populations. J. Immunol. 165, 6762–6770. 10.4049/jimmunol.165.12.6762 11120796

[B24] Kawamori T. Kaneshiro T. Okumura M. Maalouf S. Uflacker A. Bielawski J.. (2009). Role for sphingosine kinase 1 in colon carcinogenesis. FASEB J. 23, 405–414. 10.1096/fj.08-117572 18824518 PMC2630788

[B25] Kobayashi N. Kobayashi N. Yamaguchi A. Nishi T. (2009). Characterization of the ATP-dependent sphingosine 1-phosphate transporter in rat erythrocytes. J. Biol. Chem. 284, 21192–21200. 10.1074/jbc.M109.006163 19531471 PMC2755842

[B26] Lai W. Q. Melendez A. J. Leung B. P. (2011). Role of sphingosine kinase and sphingosine-1-phosphate in inflammatory arthritis. World J. Biol. Chem. 1, 321–326. 10.4331/wjbc.v1.i11.321 21537466 PMC3083938

[B27] Lee H. Deng J. Kujawski M. Yang C. Liu Y. Herrmann A.. (2011). STAT3-induced S1PR1 expression is crucial for persistent STAT3 activation in tumors. Nat. Med. 16, 1421–1428. 10.1038/nm.2250 21102457 PMC3088498

[B28] Lee H. Park H. S. Hong S. H. Choi O. K. Cho S. D. Park J.. (2013). 4-Deoxypyridoxine improves the viability of isolated pancreatic islets *ex vivo*. Islets 5, 116–121. 10.4161/isl.25254 23756681

[B29] Le Stunff H. Galve-Roperh I. Peterson C. Milstien S. Spiegel S. (2002). Sphingosine-1-phosphate phosphohydrolase in regulation of sphingolipid metabolism and apoptosis. J. Cell Biol. 158, 1039–1049. 10.1083/jcb.200203123 12235122 PMC2173216

[B30] Liang J. Nagahashi M. Kim E. Y. Harikumar K. B. Yamada A. Huang W. C.. (2013). Sphingosine-1-phosphate links persistent STAT3 activation, chronic intestinal inflammation, and development of colitis-associated cancer. Cancer Cell 23, 107–120. 10.1016/j.ccr.2012.11.013 23273921 PMC3578577

[B31] Liu X. Xiong S. L. Yi G. H. (2012). ABCA1, ABCG1, and SR-BI: Transit of HDL-associated sphingosine-1-phosphate. Clin. Chim. Acta 413, 384–390. 10.1016/j.cca.2011.11.002 22115863

[B32] Maceyka M. Harikumar K. B. Milstien S. Spiegel S. (2011). Sphingosine-1-phosphate signaling and its role in disease. Trends Cell Biol. 22, 50–60. 10.1016/j.tcb.2011.09.003 22001186 PMC3253987

[B33] Nieuwenhuis B. Lüth A. Chun J. Huwiler A. Pfeilschifter J. Schäfer-Korting M.. (2009). Involvement of the ABC-transporter ABCC1 and the sphingosine 1-phosphate receptor subtype S1P(3) in the cytoprotection of human fibroblasts by the glucocorticoid dexamethasone. J. Mol. Med. 87, 645–657. 10.1007/s00109-009-0468-x 19370318

[B34] Nopora A. Brocker T. (2002). Bcl-2 controls dendritic cell longevity *in vivo*. J. Immunol. 169, 3006–3014. 10.4049/jimmunol.169.6.3006 12218115

[B35] Olivera A. Spiegel S. (1993). Sphingosine-1-phosphate as second messenger in cell proliferation induced by PDGF and FCS mitogens. Nature 365, 557–560. 10.1038/365557a0 8413613

[B36] Ottenlinger F. Schwiebs A. Pfarr K. Wagner A. Gruner S. Mayer C.. (2016). Fingolimod targeting protein phosphatase 2A differently affects IL-33 induced IL-2 and IFN-gamma production in CD8 lymphocytes. Eur. J. Immunol 10.1002/eji.201545805 26683421

[B37] Payne S. G. Milstien S. Spiegel S. (2002). Sphingosine-1-phosphate: dual messenger functions. FEBS Lett. 531, 54–57. 10.1016/S0014-5793(02)03480-4 12401202

[B38] Price M. M. Oskeritzian C. A. Falanga Y. T. Harikumar K. B. Allegood J. C. Alvarez S. E.. (2013). A specific sphingosine kinase 1 inhibitor attenuates airway hyperresponsiveness and inflammation in a mast cell-dependent murine model of allergic asthma. J. Allergy Clin. Immunol. 131, 501.e1-511.e1. 10.1016/j.jaci.2012.07.014 22939756 PMC3563730

[B39] Pushparaj P. N. Manikandan J. Tay H. K. H'Ng S. C. Kumar S. D. Pfeilschifter J.. (2009). Sphingosine kinase 1 is pivotal for Fc epsilon RI-mediated mast cell signaling and functional responses *in vitro* and *in viv*o. J. Immunol. 183, 221–227. 10.4049/jimmunol.0803430 19542433

[B40] Saba J. D. de la Garza-Rodea A. S. (2012). S1P lyase in skeletal muscle regeneration and satellite cell activation: exposing the hidden lyase. Biochim. Biophys. Acta 1831, 167–175. 10.1016/j.bbalip.2012.06.009 22750505 PMC3609719

[B41] Schröder M. Arlt O. Schmidt H. Huwiler A. Angioni C. Pfeilschifter J. M.. (2015). Subcellular distribution of FTY720 and FTY720-phosphate in immune cells - another aspect of Fingolimod action relevant for therapeutic application. Biol. Chem. 396, 795–802. 10.1515/hsz-2014-0287 25720062

[B42] Schroeder M. Richter C. Juan M. H. S. Maltusch K. Giegold O. Quintini G.. (2011). The sphingosine kinase 1 and S1P1 axis specifically counteracts LPS-induced IL-12p70 production in immune cells of the spleen. Mol. Immunol. 48, 1139–1148. 10.1515/hsz-2014-0287 21435724

[B43] Schwalm S. Timcheva T. M. Filipenko I. Ebadi M. Hofmann L. P. Zangemeister-Wittke U.. (2015). Sphingosine kinase 2 deficiency increases proliferation and migration of renal mouse mesangial cells and fibroblasts. Biol. Chem. 396, 813–825. 10.1515/hsz-2014-0289 25781541

[B44] Siegemund S. Hartl A. von Buttlar H. Dautel F. Raue R. Freudenberg M. A.. (2009). Conventional bone marrow-derived dendritic cells contribute to toll-like receptor-independent production of alpha/beta interferon in response to inactivated parapoxvirus ovis. J. Virol. 83, 9411–9422. 10.1128/JVI.02362-08 19570869 PMC2738253

[B45] Spiegel S. Milstien S. (2007). Functions of the multifaceted family of sphingosine kinases and some close relatives. J. Biol. Chem. 282, 2125–2129. 10.1074/jbc.R600028200 17135245

[B46] Spiegel S. Milstien S. (2011). The outs and the ins of sphingosine-1-phosphate in immunity. Nat. Rev. Immunol. 11, 403–415. 10.1038/nri2974 21546914 PMC3368251

[B47] Stranges P. B. Watson J. Cooper C. J. Choisy-Rossi C. M. Stonebraker A. C. Beighton R. A.. (2007). Elimination of antigen-presenting cells and autoreactive T cells by Fas contributes to prevention of autoimmunity. Immunity 26, 629–641. 10.1016/j.immuni.2007.03.016 17509906 PMC2575811

[B48] Tabasinezhad M. Samadi N. Ghanbari P. Mohseni M. Saei A. A. Sharifi S.. (2014). Sphingosin 1-phosphate contributes in tumor progression. J. Cancer Res. Ther. 9, 556–563. 10.4103/0973-1482.126446 24518696

[B49] Van Brocklyn J. R. Lee M. J. Menzeleev R. Olivera A. Edsall L. Cuvillier O.. (1998). Dual actions of sphingosine-1-phosphate: extracellular through the Gi-coupled receptor Edg-1 and intracellular to regulate proliferation and survival. J. Cell Biol. 142, 229–240. 10.1083/jcb.142.1.229 9660876 PMC2133030

[B50] Winzler C. Rovere P. Rescigno M. Granucci F. Penna G. Adorini L.. (1997). Maturation stages of mouse dendritic cells in growth factor-dependent long-term cultures. J. Exp. Med. 185, 317–328. 10.1084/jem.185.2.317 9016880 PMC2196118

[B51] Yu H. Sun C. Argraves K. M. (2016). Periodontal inflammation and alveolar bone loss induced by *Aggregatibacter actinomycetemcomitans* is attenuated in sphingosine kinase 1-deficient mice. J. Periodont. Res. 51, 38–49. 10.1111/jre.12276 25900155 PMC4615256

[B52] Zhang L. Liu X. Zuo Z. Hao C. Ma Y. (2016). Sphingosine kinase 2 promotes colorectal cancer cell proliferation and invasion by enhancing MYC expression. Tumour Biol. [Epub ahead of print]. 10.1007/s13277-015-4700-8 26733171

[B53] Zu Heringdorf D. M. Ihlefeld K. Pfeilschifter J. (2013). Pharmacology of the sphingosine-1-phosphate signalling system. Handb. Exp. Pharmacol. 215, 239–253. 10.1007/978-3-7091-1368-4_13 23579459

